# The Effect of Real-time Headbox Adjustments on Data Quality

**DOI:** 10.16910/jemr.11.1.4

**Published:** 2018-03-21

**Authors:** Pieter Blignaut

**Affiliations:** University of the Free State Bloemfontein, South Africa

**Keywords:** Low-cost eye tracking, data quality, high framerates, head movement

## Abstract

Following a patent owned by Tobii, the framerate of a CMOS camera can be increased by reducing the size of the recording window so that it fits the eyes with minimum room to spare. The position of the recording window can be dynamically adjusted within the camera sensor area to follow the eyes as the participant moves the head. Since only a portion of the camera sensor data is communicated to the computer and processed, much higher framerates can be achieved with the same CPU and camera. Eye trackers can be expected to present data at a high speed, with good accuracy and precision, small latency and with minimal loss of data while allowing participants to behave as normally as possible. In this study, the effect of headbox adjustments in real-time is investigated with respect to the above-mentioned parameters. It was found that, for the specific camera model and tracking algorithm, one or two headbox adjustments per second, as would normally be the case during recording of human participants, could be tolerated in favour of a higher framerate. The effect of adjustment of the recording window can be reduced by using a larger recording window at the cost of the framerate.

## Introduction

This paper builds on a patent assigned to Tobii ( 14 ) to increase the framerate of a CMOS camera by reducing the size of the recording window so that it fits the eyes with minimum room to spare. The crux of the patent lies in the fact that the position of the recording window can be dynamically adjusted within the camera sensor area to follow the eyes as the participant moves the head. Since only a portion of the camera sensor data is communicated to the computer and processed, much higher framerates can be achieved with the same CPU and camera.

Besides the size of the recording window, the framerate that can be attained depends also on parameters such as the type and model of camera that is used, the amount of illumination and the focal length of the lens. The camera and lens that were used in this study allow framerates up to 350 Hz, while allowing the participant to move his head freely within a box of about 200×200 mm. More head movement can be allowed, but at the cost of framerate.

While the principle is described broadly in the said patent, no details or algorithm is provided. The principle is explained in this paper with full credit to the patent as it is available in the public domain. It is not the intention of this paper to infringe on the copyrighted patent, nor to disclose any protected intellectual property, but to evaluate the impact of the real-time adjustment of the recording window on the data quality of the eye tracker.

Since the details of implementation of the patent is not known, an algorithm to implement the process has been developed independently from the patent and is described in this paper. For purposes of the analysis, a self-assembled eye tracker with two infrared illuminators and the UI-1550LE camera from IDS Imaging ( https://en.ids-imaging.com ) was used. This equipment and the software that was developed are used for research purposes only and is not available commercially.

As a specific camera and tracking algorithm was used, the effects cannot necessarily be generalized to other trackers, but the study aims to create an awareness on the possibilities of attaining a high framerate at a low cost with acceptable data quality.

For a fixed period of time and recording at a specified framerate, a specific number of data samples should be captured. Some of these samples might, however, be lost or contain invalid data when the recording window is adjusted. The researcher needs to know the percentage of lost or invalid samples and whether the remaining samples can still be used to do valid research. The adjustments might also affect the camera latency and it is necessary to determine if, and to what extent, the accuracy and precision of the gaze data are affected by frequent head movements.

The next section will discuss the general criteria for the evaluation of eye trackers when used to analyse gaze behaviour in research projects. Subsequently, these criteria, namely data quality, freedom of head movement and tracking speed, are then discussed in more detail. The impact of the strategy to use a smaller recording window on the camera sensor and adjust it in real time to follow the eyes on the above-mentioned criteria, is evaluated by simulating head movements programmatically.

## Criteria for Evaluation of an Eye Tracker

Eye tracking can be used to obtain information on how people acquire and process information while they are reading( 37 ), browsing a Web site ( 17 ), shopping( 24 ), driving a motor vehicle( 12 ), interpreting medical images( 13 ), and performing other tasks where the ability to decipher the visual world around them is critical. Eye tracking can also be used as input modality during computer interaction, such as eye typing ( 1 ) or other gazecontingent systems( 39 ).

Regardless of the application area, the validity of research results based on eye movement analysis depend on the quality of eye movement data ( 22 ). Generally, data quality is expressed in terms of four metrics, namely *accuracy* (offset between actual and reported gaze coordinates), *precision* (reproducibility or repeatability of measures; related but not equivalent to system resolution), *latency* (time delay between occurrence of an event and the reporting thereof) and *robustness* (percentage of expected data samples that is captured) ( 22 ).

Besides good quality data, many types of research also expect gaze data to be delivered at a high speed ( 2 ). For video-based eye trackers, gaze data is based on the analysis of successive frames in a video stream and therefore the speed of an eye tracker is often referred to as its framerate. For studies where typical saccades are small and brief, as in silent reading or when studying microsaccades( 10, 27, 36 ), it is important to track at framerates in excess of 250 Hz ( 21, p. 30 ).

Many studies, such as reading or usability studies, can be done using a video-based eye tracker with participants seated in front of a computer screen. To ensure ecological validity, participants should be able to move or tilt their heads sideways, lie on their arms, lean backward, etc. as they would find comfortable( 32 ). Expecting participants to consciously keep their heads still or to put their heads in a chin rest, would deviate their attention from the task at hand and could impact on their performance.The eye tracker should, therefore, allow participants as much freedom as possible with regard to head movement.

Depending on the nature of the study, it is evident that eye trackers may be required to present data at a high speed, with good accuracy and precision, small latency and with minimal loss of data while allowing participants to behave as normally as possible. These requirements are discussed in more details below.

## Quality of Eye Tracking Data

With reference to eye tracking, the term *data quality* refers to the evaluation of the fidelity with which the continuous variation in the eye movement signal is reflected in the values measured and reported by the eye tracker ( 39 ).

### Latency

Latency is normally described as the time difference between the occurrence of an event in the visual field of the camera and the reporting thereof ( 38 ).For this study, latency is divided into two components, namely camera latency and processing time. Camera latency is the amount of time from the moment an event takes place until the image thereof arrives at the host computer. This includes camera exposure, image acquisition and transfer through the network or USB. Processing time refers to the time needed by the host computer to locate the featuresin the image and calculate and report the gaze coordinates. This is in agreement with Gibaldi, Vanegas ( 16) who found that an eye tracker's system latency results from the sum of the image acquisition latency (hardware) and the gaze computation latency (software).

For a specific camera and under specific conditions, such as light, framerate and shutter speed, the camera latency is expected to stay constant and therefore, at a set framerate, frames are expected to be delivered to the host computer at fixed intervals. Although the arrival of frames could be out of sync with the generation thereof, the phase difference is assumed to stay constant.

Repositioning of the recording window could cause a short stutter on the camera sensor, which will be propagated to the receiving end. This will manifest in a longerthan-expected interval between successive frames, which is referred to below as the delivery delay. In other words, it is unnecessary to have access to the absolute camera latency to study the effect of adjustments of the recording window as the effect on latency can be represented by the effect on the delivery delay. If the latency stays constant, the delivery delay is supposed to be zero.

### Robustness

For a fixed period of time and recording at a specified framerate, the eye tracker is expected to capture a specific number of data samples. Loss of data typically occurs when some of the critical features of the eye image – for example, the pupil and/or corneal reflection – cannot be detected reliably ( 21, p.141 ). Typically, glasses and contact lenses may cause reflections that can either obscure the corneal reflection or incorrectly be regarded by the eye tracker as being corneal reflections. Participant-related eye physiology,for example, droopy eyelids or narrow eyes,may also obscure the glint or pupil or part thereof, with subsequent loss of data. Robustness can be expressed in terms of the percentage of expected samples that are captured.

Besides the effect on latency, the short delay before frame delivery caused by repositioning of the recording window on the sensor will also result in less than the expected number of frames being delivered to the host computer. This phenomenon was confirmed by Holmqvist and Andersson ( 20, pp. 167, 168 )for an SMI RED 250 Hz eye tracker. Gaze events that occurred at the same time as adjustment of the recording window, would not be recorded.

### Accuracy

The ISO defines accuracy as the “closeness of agreement between a test result and the accepted reference value” ( 25). In layman terms, accuracy can be regarded as the offset between the actual fixation positions and the position as reported by the eye tracker.

The traditional manner of recording data for data accuracy measurements is to ask participants to focus on various small gaze targets across the display while gaze data is recorded ( 5 ). The offsets at all target positions are then averaged to obtain the accuracy of the system under the current conditions. The accuracy of a specific eye tracker is neither absolute nor constant and stating the manufacturers’ specifications only could be misleading. Accuracy depends on factors such as the hardware, ambient light, some physiological characteristics of the participants, calibration procedure, polynomial for interpolation, gaze angle, etc. ( 6, 23 ).Hansen and Ji ( 18 ) provided an overviewof remote eyetrackers and reported the accuracy ofmost model-based gaze estimation systems to be in theorder of 1°–2°.

### Precision

Precision is defined as the “closeness of agreement between independent test results obtained under stipulated conditions” ( 25 ). The spatial precisionof eye tracking data is an indication of variation of gaze data over a period of time. In other words, if the same gaze position is reported for every sample recorded by the eye tracker while a participant fixates on a target, the precision is 0. Variation in reported gaze data can originate from either system variations or human (physiological) variations.

Closely related to spatial precision is a measure termed spatial resolution, which refers to the smallest eye movement that can be detected in the data ( 22 ).

Spatial precision can be calculated as the square root of the pooled variance over the X and Y dimensions of the display (referred to as STD hereafter). In other words, precision = √((σ_x_² + σ_y_)/2) where σ_x_² = 1/N ∑^N^_i=1_ (x_i_ - ∅x)² and σ_y_² = 1/N ∑^N^_i=1_ (y_i_ - ∅y)². Another common measure of precision is the root-mean-square of the distance between subsequent points, RMS = √((∑^N^_i=1_d_i_)/N ( 22 )). This paper will report STD precision values.

## Eye Tracking and Head Movement

### Head-mounted vs remote eye trackers

Video-based eye trackers can be head-mounted or remote devices. Although head-mounted eye trackers produce more accurate results( 43 ) and allow for more head movement ( 29 ), they are intrusive. Remote devices are less intrusive and do not require any physical contact with the user, but are sensitive to head movements – especially movements in the depth dimension ( 8 ).

### Finding the point of regard

With remote eye tracking, gaze coordinates are determined through either a feature-based or appearance- or model-based approach ( 18, 26 ). Feature-based methods use the features in an eye video, i.e. pupils and corneal reflections, to map to gaze coordinates through interpolation ( 18 ). Appearance-based methods use a geometric model of the eye that can be learnt through a deep neural network, to estimate the 3D gaze direction( 27, 42 ).

For feature-based systems, head movements within the field of view of the camera can lead to reduced accuracy as the subject moves away from the calibration position ( 8, 40 ). Larger head movements would cause total loss of gaze data and affect the fixed camera latency as the camera has to be redirected again. Accuracy can be improved by alternative calibration procedures ( 8, 31 ) or by using an appearance-based approach that maps an eye image into gaze coordinates( 3, 42 ). These solutions are, however, not always feasible – as illustrated by the alternative calibration proposed by Nguyen et al. ( 3 ) that takes more than 10 minutes to perform.

Although being simpler to execute, feature-based methods are more restrictive than model-based methods in terms of permissible head movement ( 11 ). Depending on the accuracy that is required for a specific study, a small amount of head movement may be tolerated. It is customary to let a participant use a chin rest or bite bar to limit head movements if accuracy is of high importance ( 30 ).

### Requirements of the image acquisition system

To allow for free head motion, a large field of view is required ( 19 ) but to obtain high-accuracy, a video-based eye-tracker must acquire a high-resolution image of the eye ( 30, 35 ), which essentially means that the field of view must be confined ( 26 ). In general, there is a tradeoff between the field of view of an eye tracking system and the gaze estimation accuracy ( 40 ).

To maintain high accuracy with remote, feature-based systems while allowing head movement, the image acquisition system must be able to follow the eyes ( 30 ). Some of the first approaches to achieve this goal made use of multiple cameras and/or multiple illuminators ( 4, 7, 28, 33, 34, 35, 41, 43 ). Mostly these systems have one camera with a wide field of view to track head movements and another with a small field of view to track the eyes. The second camera is mechanically directed based on feedback from the first. See Hennessey et al. ( 19 )for an overview and discussion of such systems. Although this approach is effective, the mechanical nature of the set-up causes large delays and few of these systems are capable of framerates higher than 30 Hz.

Instead of using two cameras or a single stereo camera, a region of interest in a high resolution image from a single fixed camera with a wide field-of-view can be moved around to follow the eyes ( 14, 30 ). Besides being easier to set up, the redirection of the region of interest is done programmatically instead of mechanically, leading to reduced latency (quicker reaction to head movements) and improved robustness (less interruption in the stream of gaze data).

### Use of programmable CMOS cameras

CMOS (complementary metal-oxide semiconductor) cameras use active pixel sensors (APS), containing a photo detector and transistors to combine the image sensor function and image processing within the same integrated circuit ( 15 ).

CMOS cameras can be programmable with a software development kit (SDK) that enables developers to manipulate camera properties such as shutter speed, framerate and colour depth. The use of programmable CMOS image sensors provides a number of important advantages for eye tracking, *inter alia* fast sampling rates, direct pixel addressing for pre-processing and acquisition, and hard-disk storage of relevant image data ( 9 ).

Modern CMOS cameras also solve the problem of the trade-offs between accuracy and robustness on the one end and framerate to a large extent. High resolution camera sensors that provide enough clarity for the features to be found with high accuracy and precision while simultaneously allowing a wide field of view at a high framerate, are available. Alternatively, a camera with a high resolution sensor and large enough field of view, but with lower native (full sensor) framerate can be used. Only a portion of the image (the so-called recording window) can be communicated to the host computer( 14 ), which will allow the native framerate to be exceeded by several factors. The challenge with this approach is two-fold, namely to determine the optimum size of the recording window and to position it over the eyes.

The size of the recording window (RW) determines the frame rate. The smaller the RW, the higher the achievable framerate. In turn, the focal length of the lens determines the area that can fit into the RW. The higher the focal length, the better the resolution and associated accuracy, but less of the real world object can fit into it. In other words, it is desirable to have a longer focal length and a smaller recording window. Unfortunately, a tight fit of the recording window around the eyes will limit head movement to a large extent. As indicated by Elvesjo, Skogo ( 14 ), the solution lies in the adjustment of the recording window in real-time to follow the eyes without affecting latency or causing loss of frames during the adjustment.

## Experimental details

In order to examine the effect of real-time headbox adjustments on the data quality delivered by an eye tracker at various framerates, gaze data had to be recorded for a range of framerates and headbox adjustments.

For this experiment, the context was that of a stationary seated participant in front of a single computer display. The idea was that the system should compensate for smooth head movements due to the participant changing position from time to time, for example leaning sideways, moving forward/backwards, etc.

It is important to note that the study was done with a specific model of camera and a specific tracking algorithm. Although it is known that the SMI REDm, REDn, RED250 and 500, the Tobii T120, T60 and TX300 all use active recording windows, one should be careful to compare the results with them since they probably use more expensive cameras. Existing low-cost commercial eye trackers such as the Tobii EyeX ( tobiigaming.com/product/tobii-eyex/ ), Tobii 4C ( tobiigaming.com/eye-tracker-4c/) and MyGaze( www.mygaze.com) (now discontinued)deliver comparable data quality but probably don't use a smaller recording window or else they would have been able to obtain higher framerates.

### Camera and lens

An eye tracker with two infrared illuminators, 480 mm apart and the UI-1550LE camera from IDS Imaging ( https://en.ids-imaging.com ) was assembled. The UI1550LE camera with daylight cut filter has a 1600×1200 sensor with pixel size 2.8 μm and a native framerate of 18.3 fps (period = 54.6 ms).(There is a linear relationship between the number of pixels and the minimum possible interval between frames.)

The camera was fitted with a 10 mm lens from Lensation ( http://www.lensation.de/ ). Although the camera is more sophisticated than a web camera, the camera, lens and lens adapter are available from the manufacturer at about 300 euro.

The camera and lens has a field of view of 288×217 mm at 700 mm distance. A recording window of 500×116 pixels captures an image of a 90×21 mm world object at this distance which allows 198×196 mm for head movements in world space.

### Computer and Screen

Data was recorded with a desktop computer with an i7 processor and 16 GB of memory, running Windows 10. A 22 inch screen, 474×299 mm, with resolution 1680×1050, was used to display the stimuli. This means that at a gaze distance of 700 mm, 1 degree of gaze angle subtends 43 pixels (approximately 12 mm) on the display.

### Software to inspect and adjust the recording window

**Figure 1.**
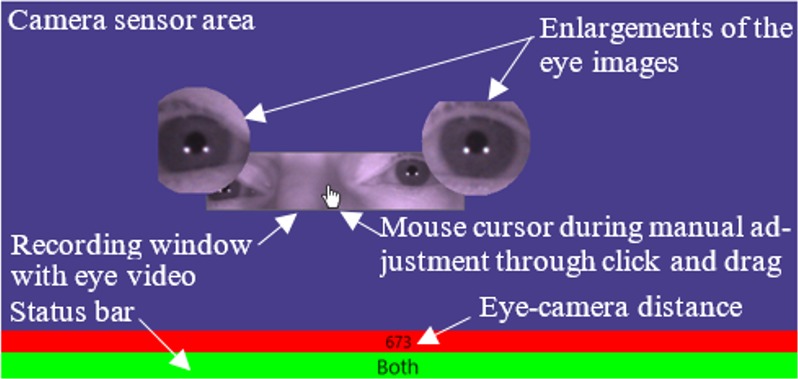
Recording window with eye video within the camera sensor area.

Software was developed using C# with .Net 4.5 along with the camera manufacturer’s software development kit (SDK) to control the camera settings and process the eye video. The software system provided an inspection panel in which the camera sensor area is represented on the computer screen, on a dark blue background (Figure 1). The portion of image data that is communicated to the PC and analysed (RW) is displayed inside this area as an eye video. Figure 1 also shows the eye-camera distance in mm and a status bar to indicate which eye(s) is/are currently inside the RW.

When a participant is seated, the recording window (RW) can be adjusted around his/her eyes manually in three ways: the chair and seating position can be adjusted, the camera can be adjusted and the RW can be grabbed with the mouse and dragged within the confines of the sensor area. Once the RW is positioned around the participant's eyes, it will be adjusted in real-time to follow smooth head movements.

### Recording window

Through the software development kit (SDK), the camera allows the selection of a smaller area of its sensor to be communicated through USB 2. The size of the recording window was chosen to (i) provide a tight fit around the eyes in order to maximise the probability of headbox adjustments for the sake of the experiment and (ii) to allow the maximum possible framerate with the specific camera model. This allowed for a worst-case scenario to investigate the effect of headbox adjustments on data quality at various framerates. This specific size is, by the way, very close to that used by the SMI RED 250 (a video clip to illustrate this can be provided when requested).

The two illuminators provided enough light for a short exposure time which, together with a recording window (RW) of 500×116 pixels, allowed a maximum framerate of 352 Hz.

The recording window fitted the image of the eyes at 700 mm gaze distance with some margin to spare (cf. Figure 1). Through the SDK, the framerate could be adjusted with automatic adjustment of the exposure and gain.

### Real-time adjustment of the recording window

**Figure 2.**
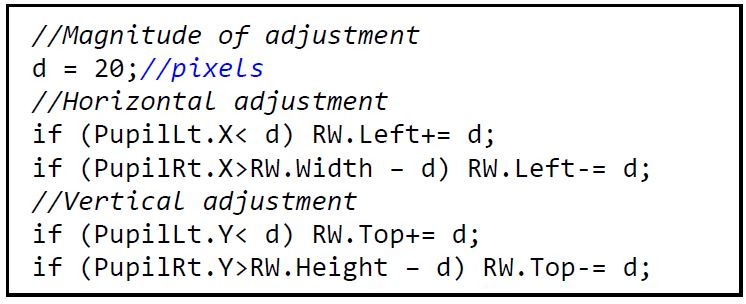
Algorithm for adjustment of the recording window in real-time. Note that the camera's mirror property is set to true so that the left edge of the sensor is displayed on the right-hand side of the image and vice versa.

The position of the eyes in the recording window is used to adjust the recording window within the headbox as the head moves around. Figure 2 shows the algorithm that is executed for every frame that is received from the camera. The magnitude of the adjustment, d, should be such that the eyes will not move outside of the RW. If d is too small, a fast or jerky movement of the head will allow the relevant eye to move out of the RW. If it is too large, the opposite eye will move out of the RW. A value of d = 20pixels (4% of the width of the recording window) worked well for smooth head movements and for the range of framerates that was tested in this study (50 Hz-350 Hz).

The eyes are only lost if the recording window cannot fit into the sensor area or if the participant suddenly jerks his head to one side. In that case, the experimenter will have to adjust the RW manually again as explained above. The software can be developed such that the sensor area and recording window (Figure 1) are visible on the experimenter's screen and that manual adjustments can be made during recording without interrupting the participant.

**Figure 3.**
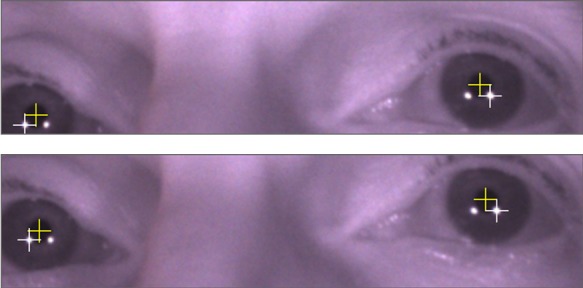
The recording window of two successive camera frames. In the first frame, the left pupil is too close to the bottom edge of the recording window. The second frame shows the recording windowafter adjustment.

Figure 3 shows two successive eye video frames at 200 Hz (5 ms interval). The crosshairs show the reported centres of the pupils and outer glints. In Frame 1, the left pupil is too close to the bottom of the recording window and needs to be corrected. In Frame 2, the recording window is adjusted so that the pupil is further from the border.

### Simulation of head movements

Instead of asking participants to move their heads from side to side or up and down, their heads were stabilised by a chin rest and head movements were programmatically simulated by adjusting the recording window instead. This procedure allowed for easier control of the amount and speed of movement for evaluation purposes.

The recording window was adjusted either horizontally or vertically by d pixels every t milliseconds. This is the same amount of adjustment if a participant would "bump" his head against one of the walls of the headbox during normal recording (cf. algorithm in Figure 2).Once the eyes get within d pixels from the edge of the recording window, every adjustment will result in an immediate correction according to the algorithm in Figure 2 – thus causing the recording window to be adjusted back and forth. At lower framerates, the two adjustments could be done within one frame, but at higher framerates it is probable that the adjustments are done in successive frames.

The value of t ranged from 100 ms to 900 ms in increments of 200 ms. This means that eleven recordings were made at each value of the framerate, namely five with vertical adjustments, five with horizontal adjustments and one with a stationary headbox.

### Data capturing

**Figure 4.**
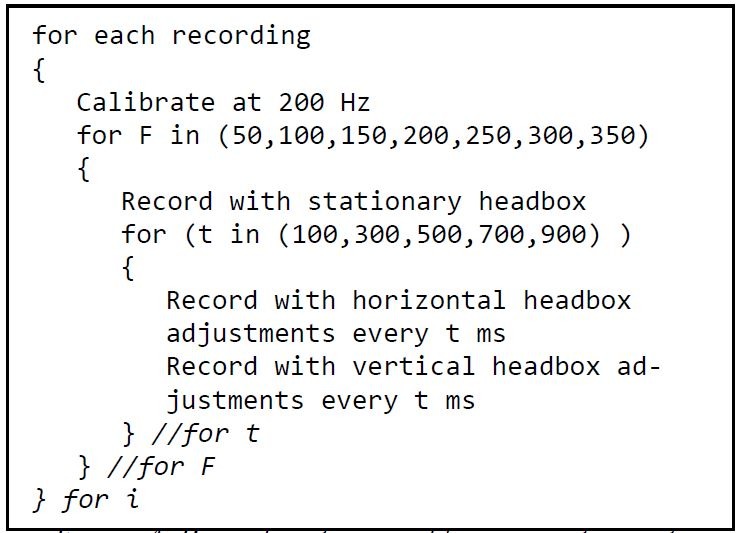
Procedure to record human participants

The recording framerates were programmatically adjusted from 50 Hz to 350 Hz with increments of 50 Hz as part of the recording procedure. Refer to Figure 4 for an algorithmic outline of the procedure that was followed.

**Figure 5.**
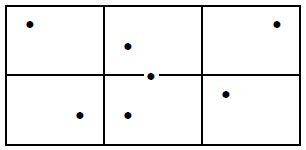
Position of gaze targets. The centre target is fixed while the others appeared in random positions within the six rectangles

Seven gaze targets (one in the middle of the display and one in a random position in each of six rectangular areas on the display (Figure 5) were displayed one at a time for each combination of framerate and headbox adjustment. Targets were displayed for 1.5 seconds each,but gaze data was recorded during the second 750 ms only to allow time for the eyes to settle on a target once it appeared. Taking into account also the time taken to save data after every set of seven targets and adjust the framerate programmatically before the next set, the recording of every set of 7 targets took about 12 s.

The above mentioned combination of gaze targets (7 values), range of framerates (7 values) and intervals of headbox adjustments (11 values) meant that 7×7×11 = 539 gaze targets had to be presented to each participant. A single recording lasted about 15 minutes. Participants had the opportunity to pause between target sets to rest their eyes if necessary.

### Participants

A participant was seated with his head stabilised by a chinrest at a distance of 700 mm in front of the camera.The chair, chinrest and camera were manually adjusted until both eyes were visible in the recording window, and the recording window was centred in the sensor area.

In order to save time and not interrupt the recording process, calibration was done before the recording commenced. Calibration was done at 200 Hz, which is in the middle of the range of framerates for which data was captured. One could argue that the calibration should have been repeated every time when the framerate changed, but that would have rendered the data capturing procedure unpractically long with an interruption every time that the framerate was changed.

The lengthy and exhausting procedure for human participants presented a challenge in terms of recruiting a large number of participants. Since the aim of the study was not to determine the absolute values for indicators of data quality for a variety of participants, but rather to compare data quality for various settings of framerate and headbox adjustments, it was decided to use only two participants (one of which was the author) and record data over a few repetitions over a period of time. This also meant improved consistency with respect to gaze behaviour over recordings.

### Artificial eyes

The aim of the study was to investigate the effect of real-time headbox adjustments on the quality of eyetracking data. In order to control for as much variance as possible due to participant-specific characteristics, data was also recorded for a set of artificial eyes. Of course, the effect of headbox adjustments on the accuracy of tracking could not be evaluated through the use of artificial eyes.

The artificial eyes were mounted at a fixed distance of 700 mm in front of the camera. The artificial eyes were aimed roughly at the centre of the screen and gaze data was recorded for seven repetitions, imitating the seven gaze targets, for all combinations of framerate and headbox adjustments. The entire procedure was repeated 10 times.

## Results

Because of the small number of participants and better control over external factors, the data from the artificial eyes were considered to be more reliable than the datafrom the human participants. However, since data was in any case recorded for human participants to facilitate analysis of accuracy, it was also analysed and presented below. Where the results for human participants do not agree with that of the artificial eyes, the conclusions are based on the results of the artificial eyes.

### The effect of headbox adjustments on delivery delay

As explained above, the effect of headbox adjustment on latency can be represented by the effect on delivery delay as the time between image acquisition and delivery (camera latency) is expected to be constant. The delivery delay can be expressed in terms of the difference between the expected between-frames interval (based on the set framerate) and the actual interval (as determined by subtracting timestamps of successive frames). In the absence of any effect on latency, the delivery delay should be zero.

Figure 6a shows the frame-to-frame interval for 6 fixations of one specific participant at 50 Hz and 100 ms between headbox adjustments. Zooming into a specific fixation revealed that instead of 20 ms between frames, one frame in every 100 ms is lost with the effect of a 40 ms gap to the next frame.

Figure 6b shows the frame-to-frame interval at 50 Hz and 500 ms between adjustments. This time, there is only one lost frame per 750 ms fixation. Because of the lower percentage of lost frames and the subsequent smaller effect on the average frame-to-frame interval, it is expected that, for the same framerate, the average delivery delay will be shorter when less frequent headbox adjustments are made.

Figure 6c shows a single fixation at 200 Hz and 100 ms between adjustments. The moment of adjustment can clearly be identified by the periodic doubling of the interframe interval – once every 100 ms. It is expected that the effect of the lost frames will be less pronounced at higher framerates as the percentage of affected frames is reduced unless more than one frame is lost for every adjustment as shown in Figure 6d for a recording at 300 Hz.

Figure 7 shows the average delivery delay for each value of the adjustment intervals and framerates that were tested for artificial eyes and human participants. Note that in this and all subsequent figures, the points on the X-axis are categorical. The points are shown with horizontal offsets to avoid overlapping (and thus improve readability) of the vertical bars that indicate the 95% confidence intervals, but these offsets do not have meaning.

**Figure 6a.**
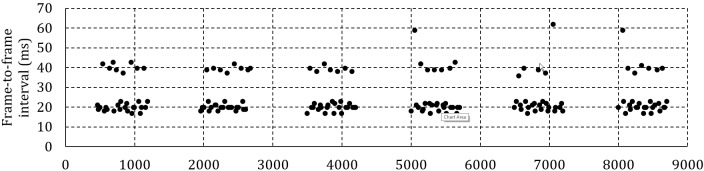
Frame-to-frame interval for a human participant at 50 Hz and 100 ms between headbox adjustments.

**Figure 6b.**
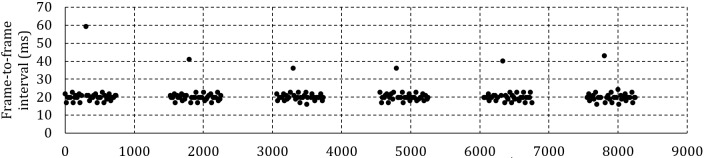
Frame-to-frame interval for a human participant at 50 Hz and 500 ms between headbox adjustments.

**Figure 6c.**
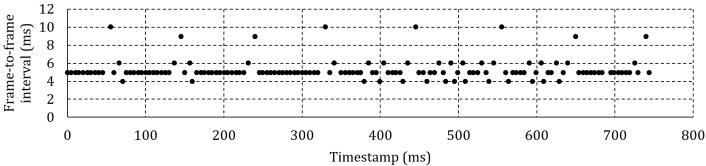
Frame-to-frame interval for one fixation at 200 Hz and 100 ms between headbox adjustments.

**Figure 6d.**
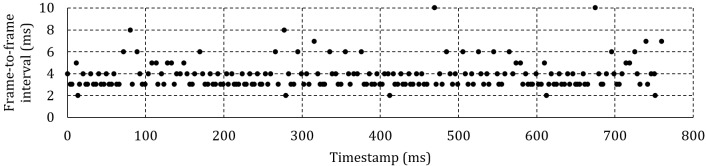
Frame-to-frame interval for one fixation at 300 Hz and 100 ms between headbox adjustments.

**Figure 7.**
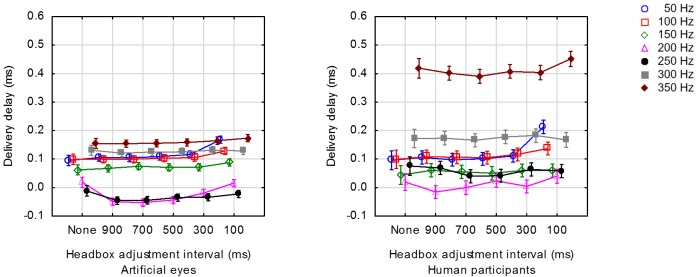
Average delivery delay for a range of framerates at 6 distinct values for the interval between headbox adjustments. Note that the points on the X-axis are categorical and are shown with horizontal offsets to avoid overlapping of the vertical bars (95% confidence intervals). These offsets do not have meaning.

The expected trend of shorter delivery delays as the framerate is increased, is confirmed for 50 Hz – 250 Hz. The inconsistency at higher framerates could possibly be attributed to the fact that the headbox was adjusted twice every t milliseconds during simulation of head movements as explained above and that the successive adjustments could not be accommodated in a single frame (cf Fig. 6d).

Table 1 shows the results of a factorial analysis of variance for the effects of the interval between headbox adjustments and framerate on delivery delay. Since the interaction between the factors was significant (α =.01), a series of separate analyses of variance was done for each of the framerates that were tested (Table 2). The entries under *post hoc* list the individual differences that were significant according to Tukey's HSD post hoc test for unequal N. As expected, a significant increase in delivery delay for headbox adjustments every 100 ms was found at the lower framerates for both artificial and human eyes.

**Table 1:** The effect of framerate and headbox adjustment interval on delivery delay (**significant at α=.01).

		Artificial eyes		Human eyes
Factor	df	F	p	F	p
Adjustment interval	5	23.953	.000**	8.499	.000**
Framerate	6	682.7	.000**	599.5	.000**
Interaction	30	3.521	.000**	2.291	.000**

**Table 2:** The effect of adjustment interval on delivery delay while controlling for framerate (*sign at α=.05; **sign at α=.01).

Artificial eyes
FPS			post hoc*					
(Hz)	F(5)	p	N	9	7	5	3	1
50	11.433	.000**	1	1	1	1	1	All
100	20.236	.000**	1	1	1	1	1	All
150	6.697	.000**	1	1	1	1	1	All
200	13.569	.000**	9,7,5	N,1	N,3,1	N,1	7,1	9,7,5,3
250	0.969	.436	-	-	-	-	-	-
300	1.323	.252	-	-	-	-	-	-
350	3.681	.003**	1	1	1		-	N,9,7

*post hoc:Individual significant (α=.05) differences according to Tukey's HSD for unequal N(N=None, 9=900 ms, 7=700 ms, 5=500 ms, 3=300 ms, 1=100 ms)

### Effect of headbox adjustments on processing time

Since processing of delivered frames is done on the host computer, processing time is expected to be independent of headbox adjustments that are done on the camera board. One would also expect the processing time to be independent of framerate as the processing time should only depend on the algorithms that are used to locate the feature points and map them to gaze coordinates.

**Figure 8.**
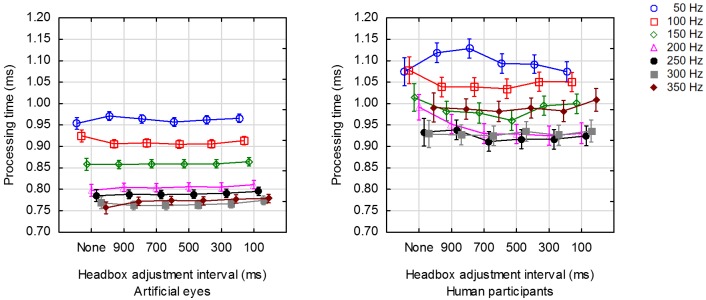
Average processing time for a range of framerates at 6 distinct values for the interval between headbox adjustments.

Figure 8 shows the average processing time for each value of the adjustment intervals and framerates that were tested for human participants and artificial eyes.

For artificial eyes, a repeated-measures analysis of variance for the effects of framerate and headbox adjustment interval on processing time indicated no interaction between these two factors (Table 3). The main effect of headbox adjustment interval was not significant (α=.05) but that of framerate was significant. There was, however, significant interaction between the factors for human participants, and therefore, a series of separate analyses of variance was done for each of the framerates that were tested (Table 4). The entries under *post hoc* list the individual differences that were significant according to Tukey's post hoc HSD test for unequal N. A significant (α=.05) effect was found for only two of the seven levels of framerate.

**Table 3:** The effect of framerate and headbox adjustment interval on processing time per frame (**significant at α=.01).

		Artificial eyes		Human eyes
Factor	df	F	p	F	p
Adj interval	5	1.986	.077	159.55	.000**
Framerate	6	1163.9	.000**	2.077	.065
Interaction	30	0.663	.919	1.505	.038*

**Table 4:** The effect of headbox adjustment interval on processing time while controlling for framerate for human participants (*significant at α=.05; **significant at α=.01).

Framerate			post hoc*					
(Hz)	F(5)	p	N	9	7	5	3	1
50	2.622	.023*	-	-	1	-	-	7
100	1.126	.345	-	-	-	-	-	-
150	2.075	.067	-	-	-	-	-	-
200	6.434	.000**	7,5,3,1	-	N	N	N	N
250	1.109	.354	-	-	-	-	-	-
300	0.129	.986	-	-	-	-	-	-
350	0.397	.851	-	-	-	-	-	-

*post hoc: Individual significant (α=.05) differences according to Tukey's HSD for unequal N (N=No adjustment, 9=900 ms, 7=700 ms, 5=500 ms, 3=300 ms, 1=100 ms)

It is not clear why processing time is less for higher framerates (cf. Figure 8). One could speculate that the operating system assigns resources (CPU time and memory) to where they are needed most and that the higher framerates attract more resources to the eye tracking application. The allocation of more and more resources can, of course, only persist until capacity is reached. For artificial eyes, the processing time stabilises at 0.75 ms – 0.8 ms for framerates of 200 Hz and higher (cf. Figure 8).

No meaning should be attached to the fact that the processing time for human eyes was higher than that for artificial eyes since the pupil sizes differed and a larger cluster of pupil pixels had to be examined for human participants. Once again, this is dependent on the specific tracking algorithm and other algorithms might produce different absolute values for processing time.

### The effect of headbox adjustments on robustness

**Figure 9.**
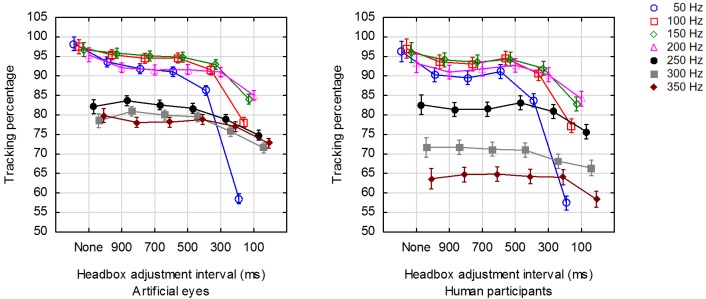
Average robustness for a range of framerates at 6 distinct values for the interval between headbox adjustments.

Figure 9 shows the average tracking percentage for each of the combinations of framerate and headbox adjustment interval for human and artificial eyes separately. Table 5 shows the results of a factorial analysis of variance for the effects of the interval between headbox adjustments and framerate on robustness. Since the interaction between the factors was significant (α=.01), a series of separate analyses of variance was done for each of the framerates that were tested (Table 6). The entries under *post hoc* list the individual differences that were significant according to Tukey's HSD post hoc test for unequal N.

**Table 5:** The effect of framerate and headbox adjustment interval on robustness (**significant at α=.01).

		Artificial eyes		Human eyes
Factor	df	F	p	F	p
Adjinterval	5	459.80	.000**	189.66	.000**
Framerate	6	582.72	.000**	727.34	.000**
Interaction	30	40.398	.000**	19.396	.000**

**Table 6:** The effect of headbox adjustment interval on robustness while controlling for framerate (**significant at α=.01).

Artificial eyes
Framerate			post hoc*					
(Hz)	F(5)	p	N	9	7	5	3	1
50	3685.1	.000**	All	All	N,9,3,1	N,9,3,1	All	All
100	1209.6	.000**	All	All	N,9,3,1	N,9,3,1	All	All
150	976.25	.000**	All	All	N,9,3,1	N,9,3,1	All	All
200	133.86	.000**	All	N,3,1	N,1	N,1	N,1	All
250	13.374	.000**	1	3,1	3,1	1	1	All
300	10.658	.000**	1	3,1	1	1	9	N,9,7,5
350	6.720	.000**	1	1	1	1	1	All
Human eyes
Framerate			post hoc*					
(Hz)	F(5)	p	N	9	7	5	3	1
50	591.60	.000**	All	N,3,1	N,3,1	N,3,1	All	All
100	326.47	.000**	All	N,3,1	N,3,1	N,3,1	All	All
150	244.10	.000**	All	N,3,1	N,3,1	N,3,1	All	All
200	55.854	.000**	9,3,1	N,5,1	1	9,3,1	N,5,1	All
250	4.082	.001**	-	1	1	1	-	9,7,5
300	2.326	.041*	-	-	-	-	-	-
350	2.222	.051	-	-	-	-	-	-

*post hoc:Individual significant (α=.05) differences according to Tukey's HSD for unequal N(N=None, 9=900 ms, 7=700 ms, 5=500 ms, 3=300 ms, 1=100 ms)

For artificial eyes, the interval between headbox adjustments proved to be a significant (α=.05) indicator of robustness for all framerates. Specifically, Tukey's posthoc comparison for the significance of individual differences revealed that the robustness when the headbox is not adjusted is significantly (α=.05) better than the robustness if the headbox was adjusted – regardless of the rate of adjustments. On the other hand, when the headbox was adjusted frequently at 10 adjustments per second, the robustness was significantly worse than when it was adjusted less frequently. This was expected as one or more frames are lost at every adjustment of the headbox.

The same trend was observed for human eyes, although the effect was not as pronounced at the higher framerates of 250 Hz, 300 Hz or 350 Hz. At the range of framerates 50 Hz – 250 Hz, the robustness for human eyes was more or less on the same level as that of artificial eyes, but at 300 Hz and 350 Hz it was considerably worse.

The results for framerate can be grouped into two clusters, namely ≤ 200 Hz and ≥ 250 Hz. Within a cluster, the robustness was more or less the same when the headbox was not adjusted often. This can again be explained in terms of the fact that only one frame was lost for adjustments at the lower framerates but more frames were lost at higher framerates (cf Figure 6d).

For framerates ≤ 200 Hz, robustness increases with framerate as the percentage of lost frames becomes less. For framerates ≥ 250 Hz, robustness decreased as more and more frames were lost for every adjustment.

In conclusion, robustness is at its best at lower framerates and with no headbox adjustments. Infrequent headbox adjustments at intervals of 300 ms or longer do, however, not affect robustness too much.

### The effect of headbox adjustments on accuracy

**Figure 10.**
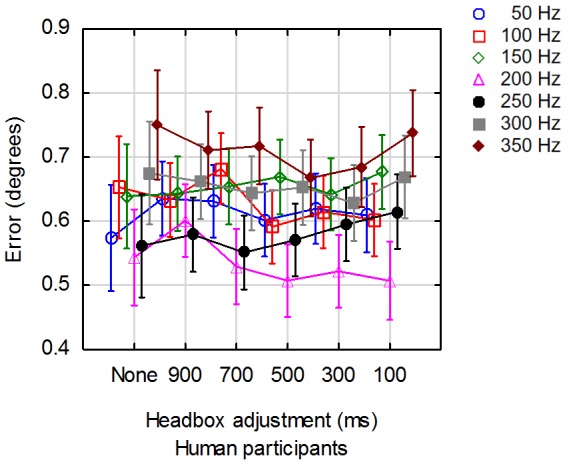
Average accuracy for a range of framerates at 6 distinct values for the interval between adjustments.

Figure 10 shows the average error for each of the combinations of framerate and headbox adjustment interval for human participants. A repeated-measures analysis of variance for the effects of framerate and headbox adjustment interval indicated no interaction between these two factors (cf. Table 7) (F(30, 4756) = 0.700, p = .888).

**Table 7:** The effect of framerate and headbox adjustment interval on accuracy(**significant at α=.01).

Factor	df	F	p
Adjustment interval	5	0.947	.449
Framerate	6	18.825	.000*
Interaction	30	0.700	.888

The main effect of framerate had a significant effect on accuracy (F(6, 4756) = 18.825, p =.000). The fact that the accuracies at 200 Hz and 250 Hz are better than the accuracies at other framerates, can probably be ascribed to the fact that participant calibration was done at 200 Hz. This was not regarded as problematic since the study focused on the effect of headbox adjustments while controlling for framerate. The researcher is also not as interested in the absolute accuracy, than in the difference in accuracy with and without headbox adjustments. In this respect, it was found that the main effect of headbox adjustment interval did not have a significant effect on accuracy (F(5, 4756) = 0.947, p = .449).

### The effect of headbox adjustments on precision

**Figure 11.**
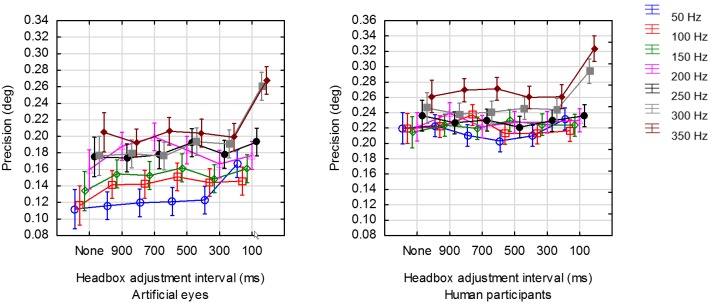
Average precision for a range of framerates at 6 distinct values for the interval between headbox adjustments.

Figure 11 shows the average STD precision for each of the combinations of framerate and headbox adjustment interval for human and artificial eyes separately. Table 8 shows the results of a factorial analysis of variance for the effects of the interval between headbox adjustments and framerate on precision. Since the interaction between the factors was significant (α=.01), a series of separate analyses of variance was done for each of the framerates that were tested (Table 9). The entries under *post hoc* list the individual differences that were significant according to Tukey's HSD post hoc test for unequal N.

**Table 8:** The effect of framerate and headbox adjustment interval on precision(**significant at α=.01).

		Artificial eyes		Human eyes
Factor	df	F	p	F	p
Adjustment interval	5	16.69	.000**	7.98	.000**
Framerate	6	67.35	.000**	39.00	.000**
Interaction	30	2.96	.000**	2.34	.000**

**Table 9:** The effect of headbox adjustment interval on precision while controlling for framerate (*significant at α=.05; **significant at α=.01).

Artificial eyes
Framerate			post hoc*					
(Hz)	F(5)	p	N	9	7	5	3	1
50	1.948	.084						
100	2.665	.021*				N		
150	2.195	.053						
200	7.814	.000**	7,9	N,3	N,3,1		9,7	7
250	1.806	.109						
300	10.863	.000**	1	1	1	1	1	N,9,7,5,3
350	7.449	.000**	1	1	1	1	1	N,9,7,5,3
Human eyes
Framerate			post hoc*					
(Hz)	F(5)	p	N	9	7	5	3	1
50	1.732	.125						
100	1.533	.177						
150	0.633	.674						
200	0.866	.504						
250	1.279	.271						
300	6.497	.000**	1	1	1	1	1	N,9,7,5,3
350	4.237	.001**	1	1	1	1	1	N,9,7,5,3

*post hoc:Individual significant (α=.05) differences according to Tukey's HSD for unequal N.(N=None, 9=900 ms, 7=700 ms, 5=500 ms, 3=300 ms, 1=100 ms.)

The interval between headbox adjustments was found to have a very significant (α=.01) effect on precision for the higher framerates of 300 Hz and 350 Hz for both artificial and human eyes. Specifically, Tukey's post-hoc comparison for the significance of individual differences revealed that when the headbox was adjusted frequently at 10 adjustments per second (100 ms intervals), the precision was significantly worse than when it was adjusted less frequently or not adjusted at all.

As expected, physiological aspects result in precision values for human participants that are slightly worse than for artificial eyes. An interesting trend was noted, namely that lower framerates lead to better precision. In other words, the best precision is obtained at lower framerates with or without infrequent headbox adjustments.

## Summary

Table 10 shows the significance of headbox adjustments on the various elements of data quality at specific framerates. For all elements but accuracy, the data captured from artificial eyes was used.

**Table 10:** Significance (α=.05) of headbox adjustments on data quality for artificial eyes at specific framerates

	Framerate (Hz)
Factor	50	100	150	200	250	300	350
Delivery delay	•	•	•	•			
Processing time							
Robustness	•	•	•	•	•	•	•
Accuracy							
Precision				•		•	•

When interpreting the results, it should be kept in mind that the absolute values of data quality is not as important as the effect of headbox adjustments on data quality. In other words, the fact that accuracy is about 0.6° should not be compared with other systems since there were only two participants for whom data is available.

The main findings are summarised per element of data quality in the following paragraphs.

### Delivery delay

Delivery delay refers to the difference in time from when a frame is expected to arrive at the host computer and when it actually arrives. A significant (α=.05) increase in delivery delay was found at the lower framerates for both artificial and human eyes when the headbox was adjusted frequently. This means that infrequent headbox adjustments, as would be the case during normal recording of human participants, will not have an effect on delivery delay.

### Processing time

Since headbox adjustments are done on the camera board and processing of frames is done on the host computer, headbox adjustments were confirmed to have an insignificant effect on processing time. For the computer that was used in this study, processing time for artificial eyes ranged from 0.75 ms at 350 Hz to 0.96 ms at 50 Hz.

### Robustness

Robustness refers to the amount of data loss during eye tracking and is expressed as a percentage in terms of the expected number of data frames for the set framerate. For artificial eyes, the tracking percentage was above 95% at framerates of 200 Hz or less when there wereno headbox adjustments. At these lower framerates, the tracking percentage dropped a little, but stayed above 90% when only one or two headbox adjustments were made per second. For higher framerates and for 3 or more adjustments per second, the tracking percentage dropped to about 70%-80%.

The robustness when the headbox was not adjusted, was significantly (α=.05) better than the robustness when the headbox was adjusted – irrespective of the rate of adjustments. On the other hand, when the headbox was adjusted frequently at 10 adjustments per second (100 ms intervals), the robustness was significantly worse than when it was adjusted less frequently.

In summary, robustness is at its best at lower framerates and with no headbox adjustments. Infrequent headbox adjustments at intervals of 300 ms or longer do, however, not affect robustness significantly.

### Accuracy

Accuracy is a measurement of the offset between actual gaze position and reported gaze position. It was found that the interval between headbox adjustments did not have a significant effect on accuracy. This should be understood against the background that robustness is affected by headbox adjustments. Calculation of point of regard is done on the host computer for received data. In other words, headbox adjustments lead to data loss, but when there is data, it is mostly accurate.

### Precision

Precision is an indication of the spread of data points around the centre. It was found that headbox adjustments affected precision quite significantly at the higher framerates of 300 Hz and 350 Hz for both artificial and human eyes, but only so when the headbox was adjusted frequently at 10 adjustments per second (100 ms intervals). At lower framerates and with less frequent headbox adjustments, STD precision for artificial eyes was in the order of 0.10°-0.14°. For human eyes, the average precision ranged between 0.20° and 0.26° as long as no more than 3 headbox adjustments were done per second.

## Conclusions

The framerate of a CMOS camera can be increased by sending only a part of the image taken by the camera through to the computer for processing. This means that the recording window must be adjusted in real-time to follow the eyes as the head moves around. The purpose of this paper was to evaluate the impact of these adjustments of the recording window on the data quality of the eye tracker.

One or two headbox adjustments per second, as would normally be the case during recording of human participants, will not have an effect on delivery delay. At a specific framerate, headbox adjustments have no effect on processing time on the host computer.

Likewise, infrequent headbox adjustments at intervals of 300 ms or longer do also not affect robustness too much. For the data that is delivered to the host computer, the accuracy will not be affected by headbox adjustments.

Headbox adjustments affect precision at the higher framerates of 300 Hz and 350 Hz for both artificial and human eyes but only so when the headbox was adjusted frequently at 10 adjustments per second (100 ms intervals).

Taking all the above into consideration, it can be concluded that a CMOS camera that allows a smaller recording window to be sent through to the host computer for processing, can be used to achieve higher framerates than is normally possible, provided that the number of adjustments of the recording window to follow the eyes in real time is limited to once or twice per second. The number of adjustments per second can be reduced by using a larger recording window, but that will have an effect on the maximum framerate that can be achieved.

## Limitations and Future Research

The results above pertain to a specific camera model and tracking algorithm. Although it is expected that the results can be generalised to even the high-end commercial trackers (see for example Holmqvist and Andersson ( 20, p. 168 )), future research should include other models and types of cameras to investigate the effect of headbox adjustments on data quality.

Specifically, the current camera does not allow segmentation of the recording window to have separate areas for the two eyes. That would have allowed even higher framerates because it would exclude the large area between the eyes which is currently also transferred to the computer and processed. Alternatively, at the same framerates, the margins around the eyes can be enlarged which would allow for a larger headbox and fewer adjustments of the recording window segments.

Furthermore, more expensive cameras would probably be less susceptible for delivery delays during adjustments of the recording window. This should be investigated.

One could also experiment with different focal lengths of the lens. A shorter focal length will reduce the size of the features and consequently enlarge the margins between the pupil and the borders of the recording window. This will allow for more head movement but probably at the cost of tracking accuracy.

The rolling shutter of the camera that was used in this study might have had an effect on the results during fast or jerky head movements. Although the focus of this paper was on the adjustment of the headbox during smooth movements, the experiments could be repeated with a camera with a global shutter.

For this experiment, the size of the recording window was fixed. A larger recording window will mean fewer headbox adjustments and will consequently have a smaller effect on data quality. Future experiments could include the size of the recording window as a factor. A generalised procedure can be established that will allow eye tracker builders to determine the smallest recording window for the specific camera model that will not affect data quality.

The adjustment algorithm in Figure 2 is based on the margins between the pupils and the edge of the recording window. The algorithm can be adapted to rather adjust the recording window such that the centroid between the pupils are centred in the window.
